# The Efficacy and Safety of Qiming Granule for Dry Eye Disease: A Systematic Review and Meta-Analysis

**DOI:** 10.3389/fphar.2020.00580

**Published:** 2020-04-30

**Authors:** Maoyi Yang, Zhipeng Hu, Rensong Yue, Liangjun Yang, Boxun Zhang, Yuan Chen

**Affiliations:** ^1^Hospital of Chengdu University of Traditional Chinese Medicine, Chengdu, China; ^2^Department of Gastroenterology, Tongde Hospital of Zhejiang Province, Hangzhou, China

**Keywords:** qiming granule, traditional Chinese medicine, dry eye disease, systematic review, meta-analysis

## Abstract

**Background:**

Dry eye disease (DED) is a common complication in clinical practice. Qiming granule, a traditional Chinese patent medicine, is widely used in treating DED in China. However, its effect is still largely unknown.

**Objectives:**

This research aims to evaluate the efficacy and safety of QG on DED.

**Methods:**

Three English database and four Chinese databases without restriction on language and publication bias were searched. Qualified literature was selecting according to inclusion and exclusion criteria, extracted the data, and conducted a meta-analysis.

**Results:**

A total of 11 articles were included in this meta-analysis. The methodological quality of included studies was low. The results showed that QG was effective for DED (RR:1.26, 95%CI:1.12 to 1.41, *P*=0.0001). The results combined with random effects model showed that QG could significantly prolong the tear film break up time (MD: 2.93, 95% CI: 2.22 to 3.65, *P* < 0.00001), increase the amount of tears in patients with DED (MD: 2.94, 95% CI: 1.83 to 4.04, *P* < 0.00001) and repair the corneal defects in patients (MD: -0.71, 95% CI: -1.25 to -0.17, *P* < 0.00001).

**Conclusions:**

This study found that despite of the apparently positive results of some outcomes, it is premature to confirm the efficacy of QG in treating DED. More high-quality studies are still needed in the future to further confirm the efficacy and safety.

## Introduction

Dry eye disease (DED) refers to a heterogeneous disease mainly involving tears and ocular surface. The occurrence and development of DED are closely related to instability and hyperosmolarity of tear film (Nelson et al., 2017), ocular-surface inflammation (Sonawane et al., 2012; Tibrewal et al., 2013), sexual hormone imbalance (Schaumberg et al., 2001; Smith et al., 2004; Uncu et al., 2006; Sriprasert et al., 2016), anatomical and neurologic disorders (Tsubota and Nakamori, 1995; Nakamori et al., 1997), compromised neural function (Stern et al., 2004; Mcmonnies, 2017), meibomian-gland dysfunction (Bron and Tiffany, 2004) and other factors like operation and medication (Fraunfelder et al., 2012; Askeroglu et al., 2013; Rosin and Bell, 2013). The clinical symptoms of DED include eye discomfort, light sensitivity, foreign-body sensation, dryness, and irritation, which can cause discomfort and fluctuating vision (Clayton, 2018).

Many therapeutic options are available for DED (American Academy of Ophthalmology (AAO) Cornea/External Disease Panel, 2013). Artificial tear drops treatment is the most widely used treatment and it can provide temporary relief of symptoms. However, according to a review, about 2/3 of patients still have symptoms despite persisting on medication (Downie and Keller, 2015). Side effects including blurred vision, variable ocular discomfort and foreign body sensation can occur (Pucker et al., 2016). Other treatment strategies including reducing inflammation, lifestyle and dietary approaches, treatment of eyelid disease and hormone therapy. However, high quality evidence is still needed to prove the efficacy of these treatments (Jones et al., 2017).

Qiming granule (QG) is a Chinese patent medicine consists of Radix Astragali (*huáng qí*), Radix Puerariae Lobatae (*gé gēn*), Radix Rehmanniae (*dì huáng*), Fructus Lycii (*g˘u q¸ z¸*), Semen Cassiae (*jué míng z¸*), Fructus Leonuri (*chōng wèi z¸*), Pollen Typhae (*pú huáng*), Hirudo (*shu¸ zh`*), which is widely used in the treatment of a variety of eye diseases in China, including diabetic retinopathy, macular edema, DED, and others (Xiangxia et al., 2009; Chao et al., 2019). A series of clinical studies suggested that QG may have a good effect on DED. However, there is no systematic summary on the clinical evidence of QG in the treatment of DED. In this study, in order to confirm the clinical efficacy of QG in the treatment of dry eye disease, the literature on the treatment of DED with QG was systematically collected, and a systematic review and meta-analysis was conducted, hoping to provide evidence-based support for clinical practice.

## Methods and Analysis

### Study Registration

This system review and meta-analysis has been registered in PROSPERO website (https://www.crd.york.ac.uk/), an international prospective system review registration website. The registration number is CRD 42018109183. The protocol of this systematic review and meta-analysis has been published (Maoyi et al., 2019). This research was conducted based on this protocol.

### Database Search

The search strategy was in accordance with Preferred Reporting Items for Systematic Reviews and Meta-Analyses (PRISMA) (Moher et al., 2009), and under the consultation with another research (Hu et al., 2019). Six databases including PubMed, Embase, Cochrane Library Central Register of Controlled Trials, China National Knowledge Infrastructure (CNKI) database, Wanfang Data Knowledge Service Platform, and the VIP information resource integration service platform (cqvip) were searched from their inception to November 2019. There were no limitations on language and publication status. In addition, conference articles and clinical registries were also searched for possible related trial. The search was conducted independently by two authors. Detailed search terms can be seen in the protocol previously published (Maoyi et al., 2019).

### Inclusion Criteria

Studies were included if they met all of the following criteria:

1. The studies design was randomized controlled trial (RCT) with parallel control group.
2. The patients in the studies must be diagnosed as DED, whether combined with other diseases or not. There is now a standardized consensus on the diagnosis of DED from TFOS DEWS II focusing on non-invasive testing (Nelson et al., 2017; Wolffsohn et al., 2017). The diagnosis of DED is based on a combination criteria of tear film break-up time test, Schimer test, corneal fluorescein staining and clinical symptoms. There is no restriction on setting of interest and other population characteristics.
3. QG was used as a treatment medicine. If other drugs were used in the treatment group, it must also be used in the control group in the same way.
4. Participants must be adults (older than 18).
5. They must include outcomes that are directly related to DED.

### Exclusion Criteria

Studies were excluded if they met any of the following criteria:

1. The study design was non RCT, such as retrospective study, cross study trial, animal study, case report, and others.
2. For multiple reports from the same study, the one with more details would be retained.
3. Without enough information about the name and use method of the study drug.
4. Papers with insufficient information to conduct a meta-analysis, such as conference papers without data, and others.

### Study Selection and Data Extraction

According to the research protocol, study search, study selection and data extraction was conducted by two reviewers independently and the third reviewer, Rensong Yue, checked the extracted data. A final decision will be made through consensus when there were discrepancies.

### Risk of Bias Assessment

The risk of bias assessment was conducted through the Cochrane collaborative bias risk tool in Review manager 5.3 software by Zhipeng Hu and Maoyi Yang. Any disagree was settled through consultation with the third author Rensong Yue.

### Statistical Analyses

Statistical analysis was mainly carried out in Review manager 5.3 software and Stata 12.0 software. The 95% confidence interval (CI) and mean difference (MD) were calculated for the continuous variables and 95% CI and risk ratio (RR) were calculated for dichotomous variables. *P* < 0.05 was considered statistically significant. The heterogeneity of data was investigated by Cochrane X^2^ and *I^2^* tests. The fixed effect model was be used if no significant heterogeneity was observed; otherwise, the random effect model would be applied for statistical analysis. Subgroup analyses were conducted to explore the source of heterogeneity. Publication bias assessment was conducted through funnel plots and Egger’s tests if more than 10 trials were included. Sensitivity analysis was used to explore the stability of the results.

## Results

### Database Search Results

**Figure 1:**
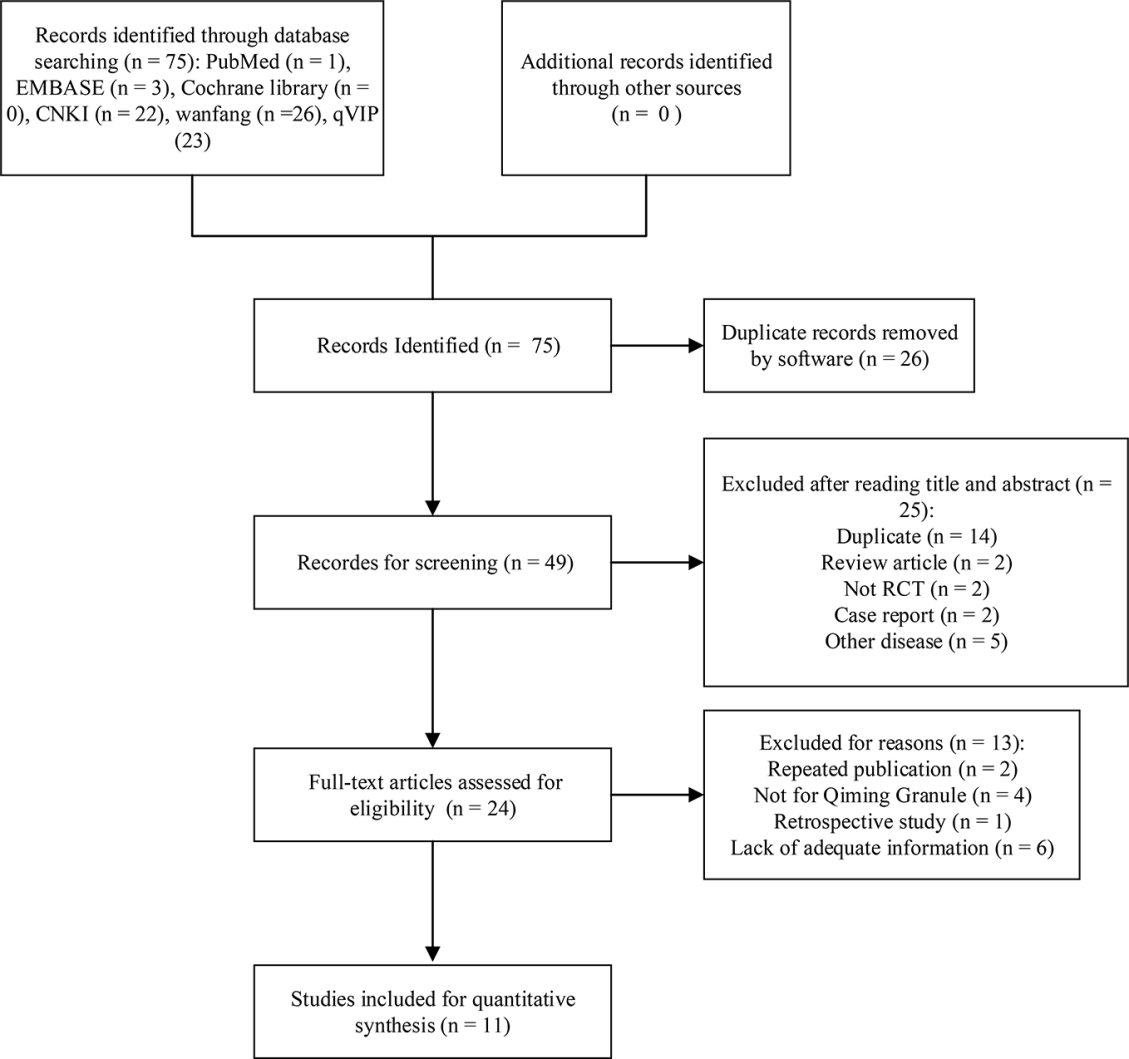
Flowchart of database searching and study identification.

A total of 75 studies have been retrieved from six databases, and no grey literature was found in the conference collection and clinical registries. 26 duplicate records were deleted by software. 25 studies were excluded by reading the title and abstract of the literature, including 14 repeatedly published literature, two retrospective studies, two non randomized controlled trials, two case reports, four studies of diseases not DED, one study of patients younger than 18 years old. The full text of the remaining 24 records were downloaded, 13 records were deleted according to the inclusion criteria or the exclusion criteria after full-text reading, and finally 11 studies were included in meta-analysis. A list of records excluded by reading the full text can be found in the **Supplementary Material 1**. The detailed process of database search is shown in **Figure 1**.

### Study Characteristics

Eleven RCTs were included in this research, all of which were completed in China (Jinlan and Mingchang, 2013; Jie and Kun, 2014; Xueling et al., 2014; Xingyan and Mingguo, 2015; Chao et al., 2016; Li et al., 2017; Shilin and Hongfang, 2017; Yi and Gaoli, 2017; Mingyue et al., 2018; Qing and Liang, 2018; Siwen, 2019). A total of 1032 DED patients were included in the study. Some studies provided information about number of eyes suffered from DED. However, these studies measured outcomes based on participants, not eyes. Therefore, the information about eyes provided by the researchers does not distort the results actually. Participants ranged in age from 34.3 to 67.6 years old. The average course of disease is 4.7 to 9.0 years. The baseline means of tear break time and Schimer’s tear test were 2.51 to 9.02 s and 2.11 to 6.64 mm, respectively. In most studies, the intervention course was one month, with four RCTs for 2 months and only one RCT for three months. In all the studies included, QG was taken orally by patients at the recommended dose of 4.5g/time, three times a day. In three studies, patients in treatment group received QG and patients in control group received conventional treatment. In the remaining eight studies, the patients in control group received conventional treatment while patients in treatment group received a combination treatment of QG and conventional treatment. Therefore, none of these studies were participants masked. The basic characteristics of the studies can be seen in **Table 1**. All the extracted information is provided in **Supplementary Material 2**. All QGs used in the included studies were prepared by and purchased from Zhejiang Wansheng Pharmaceutical Co., Ltd. None of the studies gave information about their funding and conflicts of interest. No data on quality control were reported in all the 11 studies.

**Table 1:** Characteristics of included studies.

References	Study design	Interventions		ages(treatment/control)	No. of patients (treatment/placebo)	Time of disease (treatment/placebo) (year)	Course of treatment	Outcomes	Funding information and conflict of interest
		treatment group	control group						

Siwen, 2019	RCT	QG (Qiming Granule) 4.5g, tid	sodium hyaluronate eye drops, 2–3 drops/time, tid	50.5 ± 9.2/50.5 ± 9.1	36/36	6.3±1.4/6.3±1.3	2 months	Effective rate Tear film break up time (BUT) Schimer's test	NR
Mingyue et al., 2018	RCT	QG 4.5g, tid	sodium hyaluronate eye drops, 2 drops/time, tid	37-74/39-75	105/105	1–12/2–13	2 months	Effective rate Tear film break up time (BUT) Schimer's test	NR
Qing and Liang, 2018	RCT	polyethylene glycol eye drops, tid+QG 4.5g, tid	polyethylene glycol eye drops, tid	34.3 ± 11.5/36.1 ± 15.5	60/60	NR	1 month	Effective rate Tear film break up time (BUT) Ocular surface disease index (OSDI)	NR
Shilin and Hongfang, 2017	RCT	QG 4.5g, tid	sodium hyaluronate eye drops, 2 drops/time, tid	50.5 ± 9.2/48.5 ± 9.2	20/20	1–15/1–13	2 months	Effective rate Tear film break up time (BUT) Schimer's test	NR
Yi and Gaoli, 2017	RCT	pranoprofen eye drops,1-2 drops/time, qid + artificial tears,1-2 drops/time, qid+ QG 4.5g, tid	pranoprofen eye drops,1–2 drops/time, qid + artificial tears, 1–2 drops/time, qid	67.7 ± 3.3/66.2 ± 3.3	39/39	8.9±4.1/9.0±4.1	1 month	Effective rate Tear film break up time (BUT) Corneal fluorescein staining	NR
Li et al., 2017	RCT	pranoprofen eye drops,1 drops/time, tid +sodium hyaluronate eye drops,1 drops/time, tid+QG 4.5g, tid	pranoprofen eye drops,1 drop/time, tid +sodium hyaluronate eye drops,1 drop/time, tid	52.6 ± 4.2/52.9 ± 4.5	50/45	NR	1 month	Effective rate Tear film break up time (BUT) Schimer's test	NR
Chao et al., 2016	RCT	sodium hyaluronate eye drops, 2 drops/time, qid+QG 4.5g, tid	sodium hyaluronate eye drops, 2 drops/time, qid	NR	30/30	NR	2 months	Effective rate Tear film break up time (BUT) Schimer's test Corneal fluorescein staining Eye symptoms score Zung's self-rating depression scale (SDS) Self-rating anxiety scale after treatment (SAS)	NR
Xingyan and Mingguo, 2015	RCT	pranoprofen eye drops, 2 drops/time, qid +sodium hyaluronate eye drops,2 drops/time, tid+QG 4.5g, tid	pranoprofen eye drops, 2 drops/time, qid +sodium hyaluronate eye drops, 2 drops/time, tid	57.4 ± 6.1/58.2 ± 5.8	45/37	4.9±1.9/4.8±2.1	1 month	Effective rate Tear film break up time (BUT) Schimer's test	NR
Xueling et al., 2014	RCT	pranoprofen eye drops,2 drops/time, qid +sodium hyaluronate eye drops,2 drops/time, tid+QG 4.5g, tid	pranoprofen eye drops, 2 drops/time, qid +sodium hyaluronate eye drops, 2 drops/time, tid	58.4 ± 5.9	59/56	4.7±1.8	1 month	Tear film break up time (BUT) Schimer's test	NR
Jinlan and Mingchang, 2013	RCT	Dextran and Hypromellose eye drops, qid+QG 4.5g, tid	Dextran and Hypromellose eye drops, qid	34.3/36.1	50/50	NR	3 months	Effective rate Tear film break up time (BUT) Schimer's test Corneal fluorescein staining	NR
Jie and Kun, 2014	RCT	polyethylene glycol eye drops, qid+QG 4.5g, tid	polyethylene glycol eye drops, qid	NR	30/30	NR	1 month	Effective rate Tear film break up time (BUT) Schimer's test	NR

### Risk of Bias Assessment

**Figure 2:**
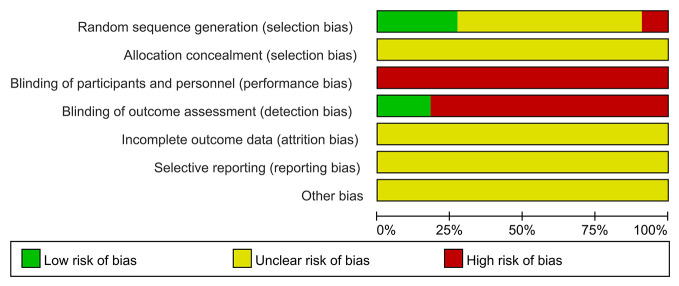
Risk of bias.

**Figure 3:**
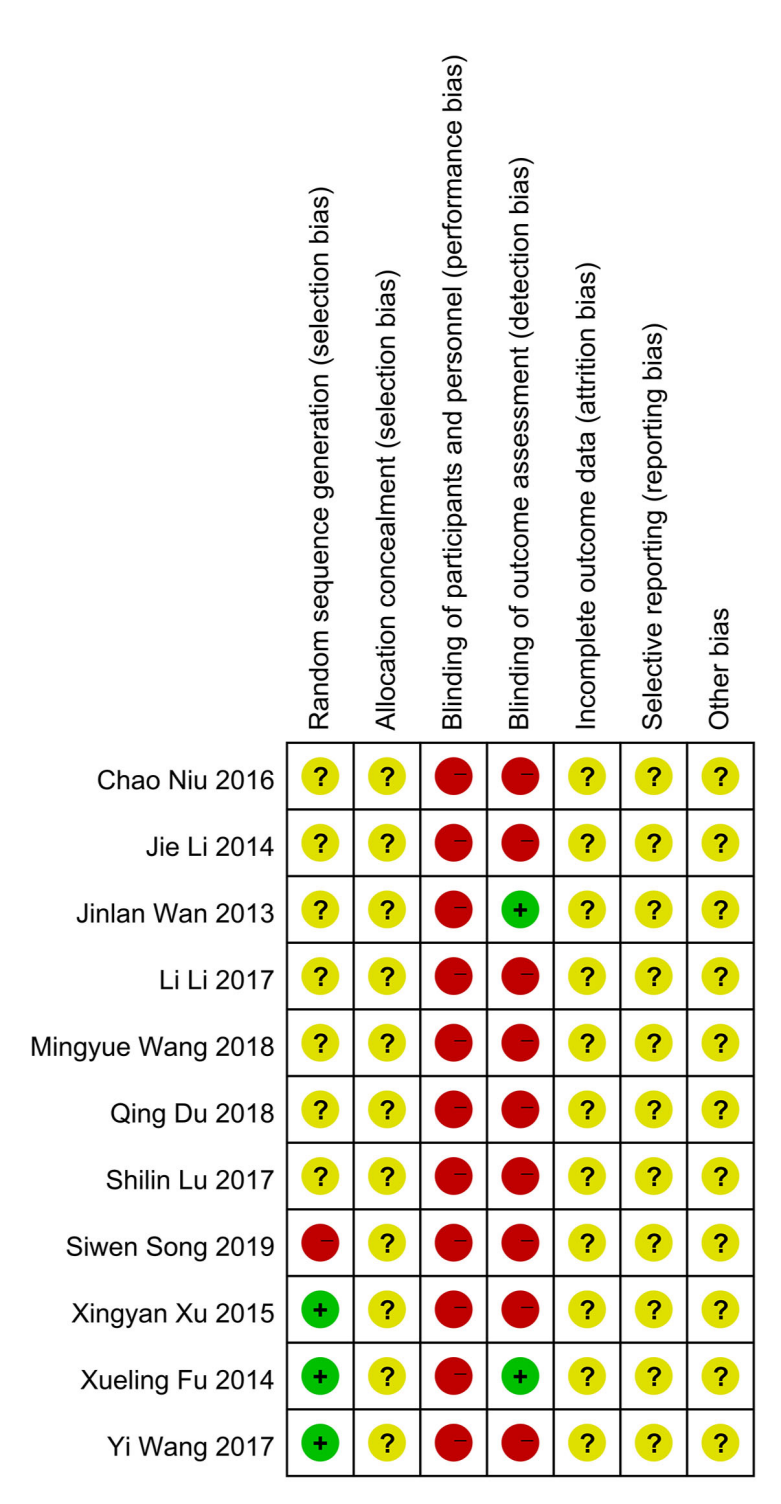
Summary of risk of bias.

In general, the quality of methodology included in the study is not high. Most of the studies did not explicitly report the method of generating random number sequence, and all of them did not clearly state whether the allocation concealment was carried out. In terms of masking method, most of the studies claimed to use blinding method, however, considering that in these experiments, patients in the experimental group received qiming granule orally and patients in the control group were given external eye drops, a high risk in terms of performance bias were determined for these studies. As to detection bias, there were two studies that use objective detection indicators, whose results would not be interfered by the detector, so these two studies were determined as “low risk” while other studies were identified as “high risk”. In the aspect of attrition bias and reporting bias, judgement about whether there was reporting bias cannot be made because all the researchers did not register their clinical trial protocol or published protocol before the trial actually started. Similarly, since all the studies had not reported the calculation of sample size in detail, both of them were marked as “unclear risk”. All risk of bias assessment data is shown in **Figures 2** and **3**.

### Total Effective Rate

**Figure 4:**
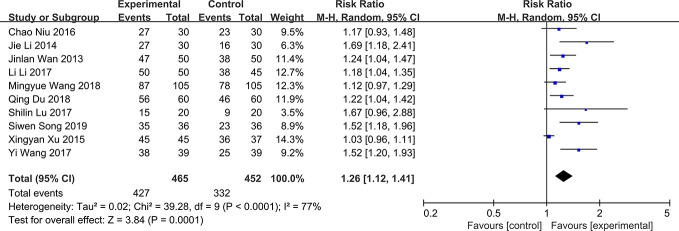
Forest plot for total effective rate.

The total effective rate is a composite end point composed of improvement of clinical symptoms, tear film break up time, Schimer’s test and corneal fluorescein staining. The results can be divided into three categories: significantly effective, effective and ineffective. There are some slight differences in the composition of this outcome in different studies. For example, other outcomes may be added to this composite end point in some studies. All the 11 RCTs included in the study compared the total effective rate of QG in DED patients. The results showed that there was significant heterogeneity in total effective rate (*P* < 0.0001, *I*^2 =^ 77%) (**Figure 4**). The results showed that QG was effective for DED (RR:1.26, 95%CI:1.12 to 1.41, *P*=0.0001) (**Figure 4**). Sensitivity analysis indicated that the result was stable (**Supplementary Material 3**). Subgroup analysis was conducted according to the course of treatment (1 month, 2 months, or 3 months) (**Supplementary Material 4**) and the intervention (qiming granule alone or combined with other drugs) of the treatment group (**Supplementary Material 5**). The results of these two subgroup analyses showed that the difference between courses of treatment and concomitant drugs did not lead to heterogeneity and the effective rate of QG would not be different because different courses of treatment and combination of drugs (test for subgroup differences: *P* all > 0.05) (**Table 2**).

**Table 2:** Subgroup analysis for outcomes.

	Number of comparisons	Results	*P* value for overall effect	*I^2^*	*P* value for subgroup difference
**Total effective rate**		Risk ratio (95% CI)			
All comparisons	10	1.26 (1.12, 1.41)	0.0001	77%	
Course of treatment					0.98
1 month	5	1.27 (1.03, 1.56)	0.02	89%	
2 months	4	1.26 (1.06, 1.50)	0.008	49%	
3 months	1	1.24 (1.04, 1.47)	0.01	NA	
Intervention of treatment group					0.63
Monotherapy	3	1.34 (1.02, 1.74)	0.03	66%	
Combined	7	1.24 (1.07, 1.43)	0.003	82%	
**Tear Film Break Up Time**		Mean difference (95% CI)			
All comparisons	11	3.84 (2.91, 4.76)	<0.00001	65%	
Course of treatment					0.0001
1 month	6	2.59 (2.09, 3.09)	<0.00001	39%	
2 months	4	3.84 (2.91, 4.76)	<0.00001	65%	
3 months	1	1.12 (0.26, 1.98)	0.01	NA	
Intervention of treatment group					0.23
Monotherapy	3	3.49 (2.85, 4.13)	<0.00001	0%	
Combined	8	2.81 (1.92, 3.71)	<0.00001	87%	
Baseline value					0.98
High baseline	4	2.90 (2.09, 3.71)	<0.00001	32%	
Low baseline	7	2.92 (1.92, 3.91)	<0.00001	89%	
**Schimer’s Test**		Mean difference (95% CI)			
All comparisons	9	2.94 (1.83, 4.04)	<0.00001	95%	
Course of treatment					0.0002
1 month	4	1.76 (1.11, 2.41)	<0.00001	27%	
2 months	4	4.28 (3.27, 5.29)	<0.00001	90%	
3 months	1	2.81 (1.24, 3.12)	<0.00001	NA	
Intervention of treatment group					<0.0001
Monotherapy	3	5.00 (4.73, 5.27)	<0.00001	0%	
Combined	6	1.98 (1.55, 2.40)	<0.00001	9%	
Baseline value					<0.00001
High baseline	3	5.07 (4.73, 5.27)	<0.00001	0%	
Low baseline	6	1.97 (1.53, 2.42)	<0.00001	9%	

NA, not appliable

### Tear Film Break Up Time

**Figure 5:**
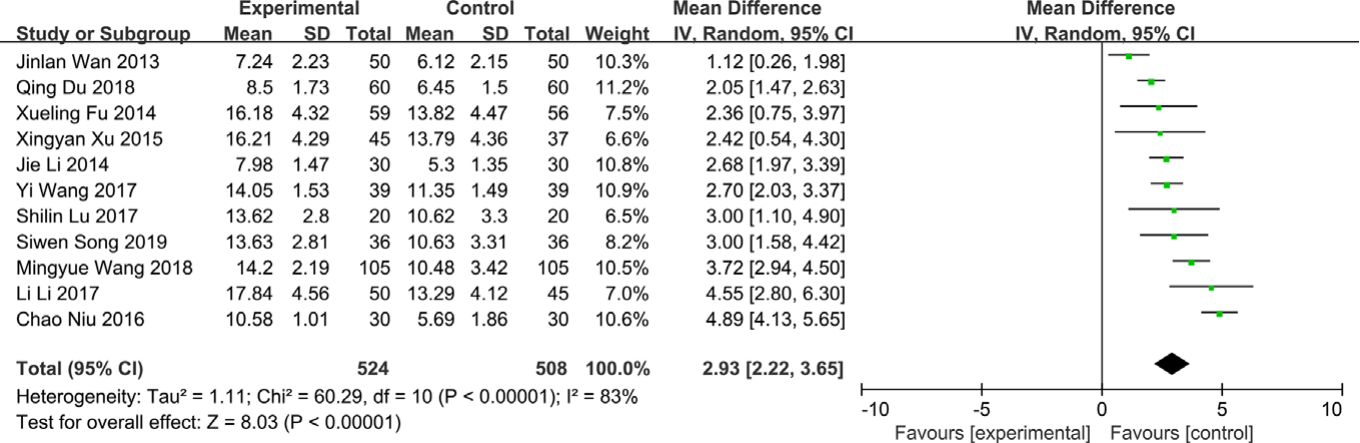
Forest plot for tear film break up time.

All the included articles studied the effect of QG on tear film break up time in patients with DED. The results showed significant heterogeneity (*P* < 0.00001, *I*^2^ = 83%) (**Figure 5**). The results combined with random effects model showed that QG could significantly prolong the tear film break up time (MD: 2.93, 95% CI: 2.22 to 3.65, *P* < 0.00001) (**Figure 5**). Sensitivity analysis indicates that the result is robust (**Supplementary Material 6**). Subgroup analyses were performed according to different course of treatment (**Supplementary Material 7**), combination of drugs (**Supplementary Material 8**), and different baseline levels of tear film break time (**Supplementary Material 9**). The results of subgroup analysis showed that the effect of QG on prolonging tear film break up time could not be affected by whether patients were treated with QG alone or combined with other drugs and by different baseline levels of tear film break up time (test for subgroup differences: *P* all > 0.05). However, different course of treatment could affect the effect of QG on the prolongation of tear film break up time (Test for subgroup differences: *P*=0.0001) (**Table 2**).

### Schimer’s Test

**Figure 6:**
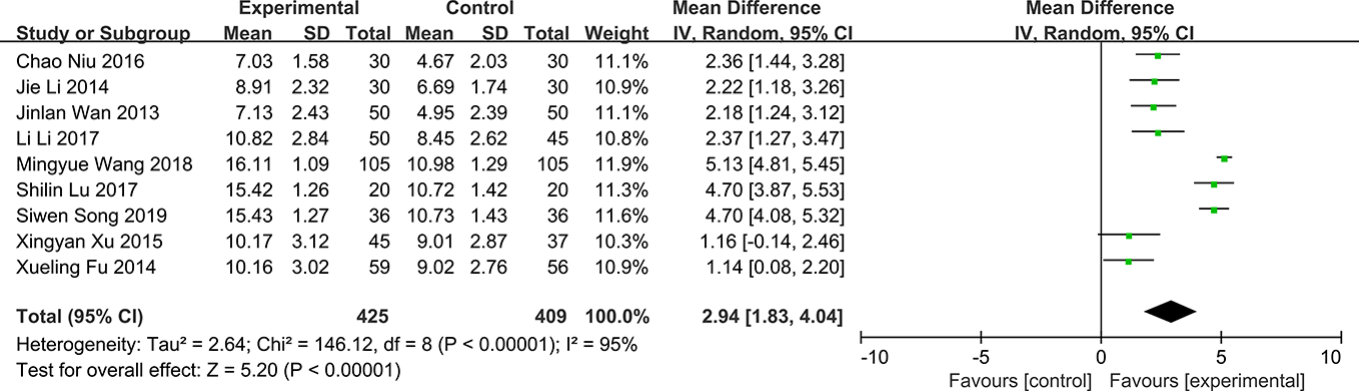
Forest plot for Schimer’s test.

Nine of the included RCTs reported the results of QG on Schimer’s test (Jinlan and Mingchang, 2013; Xueling et al., 2014; Jie and Kun, 2014; Xingyan and Mingguo, 2015; Chao et al., 2016; Li et al., 2017; Shilin and Hongfang, 2017; Mingyue et al., 2018; Siwen, 2019). There was significant heterogeneity in the results of schimer’s test (*P* < 0.0001, *I*^2 =^ 95%) (**Figure 6**). The results showed that QG could significantly increase the amount of tears in patients with DED (MD: 2.94, 95% CI: 1.83 to 4.04, *P* < 0.00001) (**Figure 6**). Sensitivity analysis indicates that the result is stable (**Supplementary Material 10**). Subgroup analyses were conducted according to three subgroup hypotheses. The results of subgroup analyses indicated that different courses of treatment (**Supplementary Material 11**), combination therapies (**Supplementary Material 12**) and baseline levels (**Supplementary Material 13**) would affect the results of Schimer’s test (test for subgroup differences: *P* all < 0.05). Comparing the results of different subgroups, it was found that the subgroups with two months of treatment, single use of QG and higher baseline level of patients had better therapeutic effect (**Table 2**).

### Corneal Fluorescein Staining

**Figure 7:**
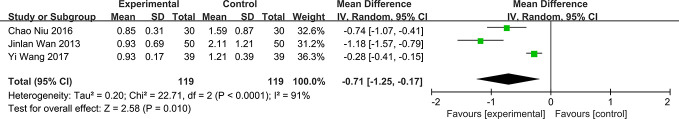
Forest plot for corneal fluorescein staining.

Among the RCTs included, three studies reported the effect of QG on corneal fluorescein staining in patients with DED (Jinlan and Mingchang, 2013; Chao et al., 2016; Yi and Gaoli, 2017). The results show that QG can significantly repair the corneal defects in patients (MD: -0.71, 95% CI: -1.25 to -0.17, *P* < 0.00001) (**Figure 7**). However, the results of corneal fluorescein staining showed significant heterogeneity in all studies (*P* < 0.0001, *I*^2 =^ 91%) (**Figure 7**). Sensitivity analysis indicates that the result is stable (**Supplementary Material 14**).

### Other Results

Two studies (Chao et al., 2016; Qing and Liang, 2018) reported the effect of QG on the ocular symptoms. One study (Chao et al., 2016) found that ocular symptom scores of DED patients were significantly alleviated (*P* < 0.05) and the other (Qing and Liang, 2018) found a significant reduction in the index of ocular surface. One study reported the effect of QG on the mental state of patients with DED (Chao et al., 2016). The therapeutic effect of QG on the mental state of patients with DED was studied by using Zung’s self-rating depression scale (SDS) and self-rating anxiety scale after treatment (SAS). The results showed that QG could improve the tension and anxiety of patients (*P* all < 0.05). One study reported the effects of QG on the levels of MMP-9 (Qing and Liang, 2018). The results showed that QG could significantly improve the level of MMP-9 in tear film and the index of ocular surface disease in patients with dry eye disease.

### Publication Bias

Funnel plot showed the published bias of effective rate (**Supplementary Material 15**) and tear film break up time (**Supplementary Material 16**). The funnel plot of effective rate was asymmetric, and the funnel plot of tear film break time was symmetric, which means that there was a significant publication bias between the results of effective rate, while there was no significant publication bias between the results of tear film break up time. The results of egger’s test were consistent with funnel plot (*P* = 0.005 and 0.117, respectively). Because the number of RCTs included in Schimer’s test was less than 10, there was no funnel chart analysis for this part of data.

### Adverse Events

Of the 11 studies included, two studies mentioned that no obvious adverse events were found during the research (Shilin and Hongfang, 2017; Yi and Gaoli, 2017), while the other nine RCTs did not mention whether any adverse events occurred (Jinlan and Mingchang, 2013; Jie and Kun, 2014; Xueling et al., 2014; Xingyan and Mingguo, 2015; Chao et al., 2016; Li et al., 2017; Mingyue et al., 2018; Qing and Liang, 2018; Siwen, 2019).

## Discussion

In this study, the clinical evidence of QG in the treatment of DED was systemically collated and analyzed so as to provide a better guidance for clinical practice. The analysis of 11 RCTs, indicated that QG can alleviate the clinical symptoms, prolong the break up time of tear film, promote the secretion of tear and repair the damaged cornea, and may have a relieving effect on the depression, and anxiety emotion of patients. However, since there is no effective control in any of the studies, we still cannot rule out whether the efficacy of QG is caused by the placebo effect. Sensitivity analysis showed that the results were stable, but the results were heterogeneous, and there was publication bias in effective rate.

The results of this study have significant heterogeneity and the source of heterogeneity was explored by subgroup analysis. The results showed that different intervention courses would affect the effect of QG on improving tear film break up time. The subgroup analyses of the Schimer’s test indicated that the heterogeneity may be caused by the course of intervention, the combination of drugs or not, and the baseline level of patients. Subgroup analyses of other outcomes failed to find out the source of heterogeneity. Therefore, pre-set subgroup hypotheses did not fully explain the heterogeneity of this study. In terms of effective rate, the scales used in 11 studies were not completely unified, which may be one of the reasons for heterogeneity. There are primary and secondary causes of dry eye disease and the causes of DED in 11 RCTs were different, which may bring heterogeneity to this study. In this study, 11 RCTs were all single center studies, so the regional differences of patients may also be one of the sources of heterogeneity. In addition, there were some defects in the trial design of the included studies, such as no placebo control, no masking method and allocation concealment, and no supervisor to supervise the quality of the trial. All of these factors may lead to the heterogeneity between studies.

DED is mainly caused by damage of corneal surface and decreased tear secretion, and manifested as eye discomfort accompanied by dryness, irritation and fluctuating or blurry vision (Higuchi et al., 2016; Mojzis et al., 2016; Park et al., 2016). Therefore, the time of tear break up and the amount of tear secretion can directly reflect the severity of the disease, so these outcomes are often used in clinical trials. This study found that QG can significantly improve tear break up time and the amount of tear, which suggested that QG can be used in patients with shortened time of tear break up and decreased amount of tear secretion (Kim et al., 2016; Sharma et al., 2016). Previous studies reported that QG was helpful for the treatment of patients with dry eye and corneal epithelial injury which was confirmed in this research. The results showed that QG could repair the damaged cornea of patients with DED. Animal studies had confirmed that QG could increase visual function, protect retinal capillary (Sha, 2013), reduce the concentration of VEGF (Ziming and Bole, 2013) and HIF-1a in retinal tissue, improve the expression level of PEDF protein in retinal tissue, and inhibit the activity of Shh pathway (Lin et al., 2013). This may be the mechanism(s) of QG in repairing corneal injury. However, the mechanism(s) of QG still needs further research.

There are some limitations in this meta-analysis. This study included a small number of RCTs and all of these RCTs were single center, small sample studies. The methodological quality of these 11 RCTs is not high, which leads to the low level of evidence in this study. None of the studies have an effective control, which may affect the judgement of efficacy. More importantly, since DED is defined by both symptoms and signs, it is critical to analyze the improvement of both symptoms and signs. In the trial included in this study, only one trial analyzed the improvement of patients’ symptoms and signs at the same time, so there is still no definite evidence to fully prove the effectiveness of QG in treating DED. In future research, more attention should be paid to development of core outcome set of DED (Wolffsohn et al., 2017).

Both the risk of bias in included studies and risk of bias due to missing results may be influenced by conflicts of interest of study investigators or funders. In this study, all included studies did not clearly report their sources of funding, so judgement about whether there were conflicts of interest cannot be made. These potential conflicts of interest may be the reason of publication bias. In the future research, researchers should report their sources of funding and conflicts of interest more clearly, which will be helpful for assessing risk of bias and exploring heterogeneity.

To sum up, QG seems to be effective in improving total effective rate, tear film break up time, schimer’s test, corneal fluorescein staining and other outcomes, but there are some defects in the methodological quality currently included and clinician should be cautious when recommending QG in treating DED. More high-quality research is needed to provide high quality evidence for further support of its efficacy.

## Conclusion

This study found that despite of the apparently positive results of some outcomes, it is premature to confirm the efficacy of QG in treating DED. More high-quality studies are still needed in the future to further investigate the efficacy and safety.

## Author Contributions

The protocol was designed by MY and ZH under the guidance of RY. This work was conducted by MY, ZH, LY, BZ and YC. The manuscript was drafted by MY and revised by RY and LY. All authors approved the final manuscript before submission. MY and ZH contributed equally to this work and should be regarded as co-first authors.

## Funding

This project was funded by the National Natural Science Foundation of China (No.81774279) and National Science and Technology Planning Project (CN) (2014BAI10B00). The sponsors are not involved in design, execution, or writing the study.

## Conflict of Interest

The authors declare that the research was conducted in the absence of any commercial or financial relationships that could be construed as a potential conflict of interest.

## Supplementary Material

Supplementary material 1 The list of records excluded by reading the full text.

Supplementary material 2 The basic characteristics of the studies.

Supplementary material 3 Sensitivity analysis for total effective rate.

Supplementary material 4 Subgroup analysis according to the course of treatment for total effective rate.

Supplementary material 5 Subgroup analysis according to the intervention of the treatment group for total effective rate.

Supplementary material 6 Sensitivity analysis for tear film break up time.

Supplementary material 7 Subgroup analysis according to the course of treatment for tear film break up time.

Supplementary material 8 Subgroup analysis according to the intervention of the treatment group for tear film break up time.

Supplementary material 9 Subgroup analysis according to different baseline levels of tear film break time.

Supplementary material 10 Sensitivity analysis for schimer’s test.

Supplementary material 11 Subgroup analysis according to the course of treatment for schimer’s test.

Supplementary material 12 Subgroup analysis according to the intervention of the treatment group for schimer’s test.

Supplementary material 13 Subgroup analysis according to different baseline levels of schimer’s test.

Supplementary material 14 Sensitivity analysis for corneal fluorescein staining.

Supplementary material 15 Funnel plot for effective rate.

Supplementary material 16 Funnel plot for tear film break up time.

## Abbreviations

DED, Dry eye disease; QG, Qiming Granule; PRISMA, Preferred Reporting Items for Systematic Reviews and Meta-Analyses; CNKI, China National Knowledge Infrastructure; cqvip, VIP information resource integration service platform; RCT, randomized controlled trial; CI, confidence interval; MD, mean difference; RR, risk ratio
