# Corrigendum: The Efficacy and Safety of Qiming Granule for Dry Eye Disease: A Systematic Review and Meta-Analysis

**DOI:** 10.3389/fphar.2020.597639

**Published:** 2020-12-11

**Authors:** Maoyi Yang, Zhipeng Hu, Rensong Yue, Liangjun Yang, Boxun Zhang, Yuan Chen

**Affiliations:** ^1^ Hospital of Chengdu University of Traditional Chinese Medicine, Chengdu, China; ^2^ Department of Gastroenterology, Tongde Hospital of Zhejiang Province, Hangzhou, China

**Keywords:** qiming granule, traditional Chinese medicine, dry eye disease, systematic review, meta-analysis

In the original article, there was a mistake in Figure 5 as published. There was a mistake in the generation of this figure, which may make this figure confusing. Some studies were unintentionally unchecked by us when generating forest plot by software Review Manager, resulting in the effect estimates and confidence intervals not being shown. Correspondingly, there are no blocks at the point estimates of intervention effect and horizontal lines of these studies in the forest plot. The corrected Figure 5 appears below.

**FIGURE 5:**
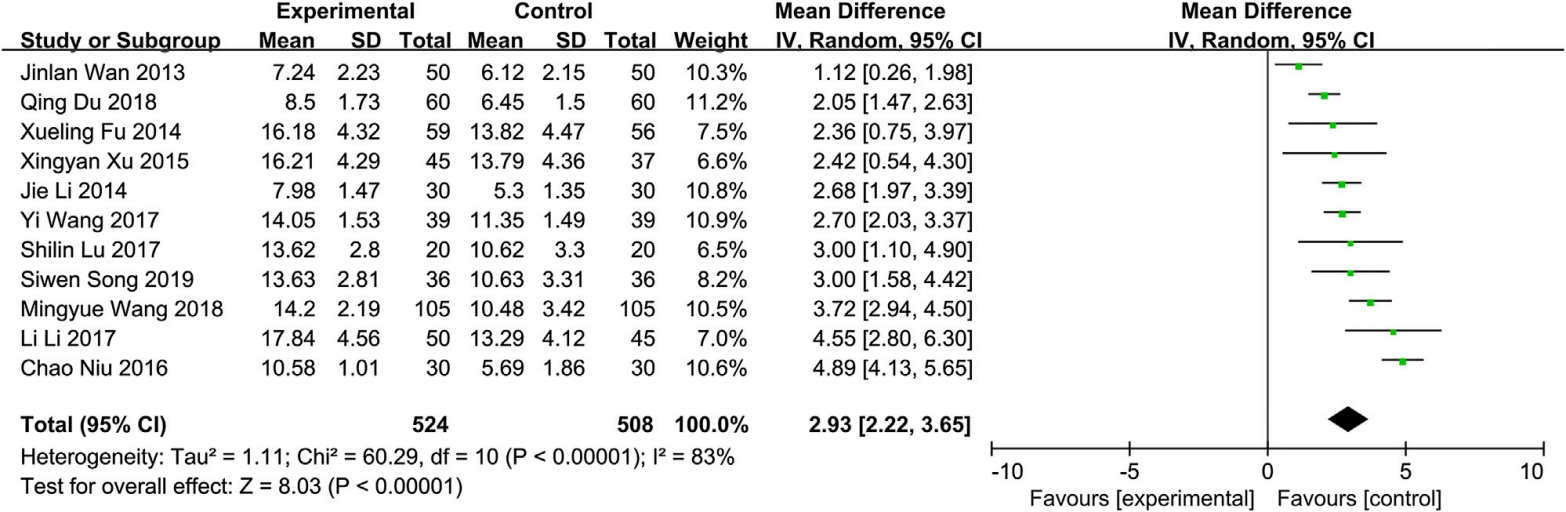
Forest plot for tear film break up time.

The authors apologize for this error and state that this does not change the scientific conclusions of the article in any way. The original article has been updated.

