# Supplementary material for: The Efficacy and Safety of Qiming Granule for Dry Eye Disease: A Systematic Review and Meta-Analysis
Source: Front Pharmacol. 2020 Apr 30;11:580. doi: 10.3389/fphar.2020.00580 (PMC7204435; doi:10.3389/fphar.2020.00580)
Supplement: Supplementary material 1 — The list of records excluded by reading the full text. [file DataSheet_1.zip › supplementary materials/supplementary material 1.docx]

**A list of excluded studies by reading full text.**

**Repeated publication：**

Wan Jinlan, Zhang Mingchang. 2013. Clinical observation of Qiming granule combined with artificial tear in the treatment of dry eye. International Journal of Ophthalmology. 13:1925-1926. doi: CNKI:SUN:GJYK.0.2013-09-067.

Xu Xyan, Lin Mingguo. 2015. Analysis of the effect of pramiphene and sodium hyaluronate combined with traditional Chinese medicine on the sequelae of hypermastia in elderly patients with diabetes mellitus. International Journal of Ophthalmology. 15:2174-2176. Doi: 10.3980/j.issn.1672-5123.2015.12.42.

**Not for Qiming Granule:**

Fu Xueling, Wang Jinduo, Jiang Ying, Liu Linying, Li Liming. 2015. Clinical observation of Qiming granule combined with pramiphene in the treatment of dry eye after hypermastia. Chinese medical emergencies. 24:2219-2221.doi: CNKI:SUN:ZYJZ.0.2015-12-053.

Li Xiujuan. 2017. Clinical research on the treatment of diabetic xerophthalmia based on syndrome differentiation of Qiming granules [Master]. Chengdu University of traditional Chinese medicine. doi: CNKI:CDMD:2.1018.954229.

Wang Feng. 2012b. Clinical study on the treatment of dry eye in patients with type 2 diabetes by traditional Chinese and Western medicine. Journal of Taishan Medical College. 33:445-446. doi: CNKI:SUN:TSYX.0.2012-06-018.

Zhu Yanni, Kou Zhangfeng, fan Weijun, Zhu Yanmei. 2018. The effect of praluofen eye drops combined with Qiming granules on tear film stability and ocular surface function in juvenile patients with dry eye after LASIK. Journal of modern Chinese and Western medicine. 27:2688-2690. doi: CNKI:SUN:XDJH.0.2018-24-021

**Retrospective study:**

Li Wei, Xu Xiaohong, Sun Nan, Peng Qi. 2019. Qiming granule combined with Deproteinized Calfblood Extract Eye Gel in the treatment of dry eye after diabetes super emulsification. International ophthalmology.19:1435-1438.

**Lack of adequate information:**

Wang Feng. 2012a. Clinical observation of Qiming granule in the treatment of dry eye in patients with type 2 diabetes mellitus. Chinese community physician (Medical Major). 14:206-207. doi: 10.3969/j.issn.1007-614x.2012.20.197.

Zhang shihong, yao xuehui, all red, et al. Qiming particles combined with hydroxyl indican eye drops to treat dry eyes [J]. Essential health (mid-month issue), 2012, 011 (007) : 280-281.

Zhao Zhihua, fan Fang, Ma Qingmin, Zhao Xiaobin. 2018. Application of Qiming granules can improve dry eye after eye surgery in T2DM patients. Genomics and applied biology. 37:80-86.

Zhao Zhihua, Li Kejun, Zhao Xiaobin, Ma Qingmin, Jia Zhimin. 2017. Effect of Qiming granule combined with artificial tear and non-steroidal anti-inflammatory medicine eye drops on corneal epithelium injury in patients with dry eye after phacoemulsification. World TCM. 12:2971-2974. doi: 10.3969/j.issn.1673-7202.2017.12.027.

Zhou yuncui. 2017. Analysis of the effectiveness of Qiming granule assisted with sodium hyaluronate in the treatment of dry eye. Inner Mongolia traditional Chinese medicine. 36:34-35. doi: CNKI:SUN:NZYY.0.2017-07-033.

Zong Zhifeng. 2018. Observation on the clinical effect of Qiming Granule on dry eye in patients with type 2 diabetes mellitus. Chongqing Medical Journal. 47.doi: 10.3969/j.issn.1007-614x.2012.20.197.
