# Supplementary figures and images for: The Efficacy and Safety of Qiming Granule for Dry Eye Disease: A Systematic Review and Meta-Analysis
Source: Front Pharmacol. 2020 Apr 30;11:580. doi: 10.3389/fphar.2020.00580 (PMC7204435; doi:10.3389/fphar.2020.00580)

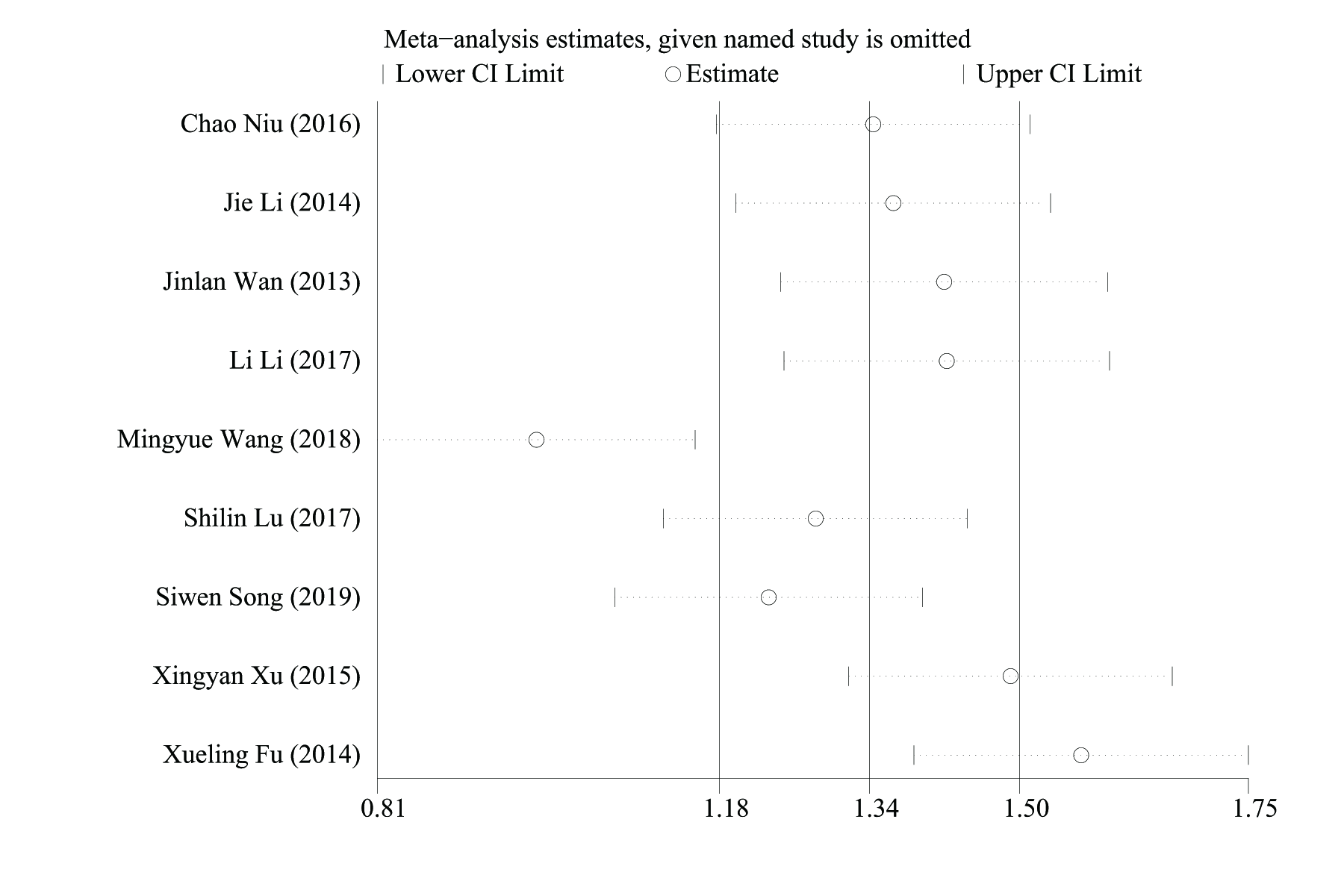

Supplement: Supplementary material 1 — The list of records excluded by reading the full text. [file DataSheet_1.zip › supplementary materials/Supplementary material 10.tif]

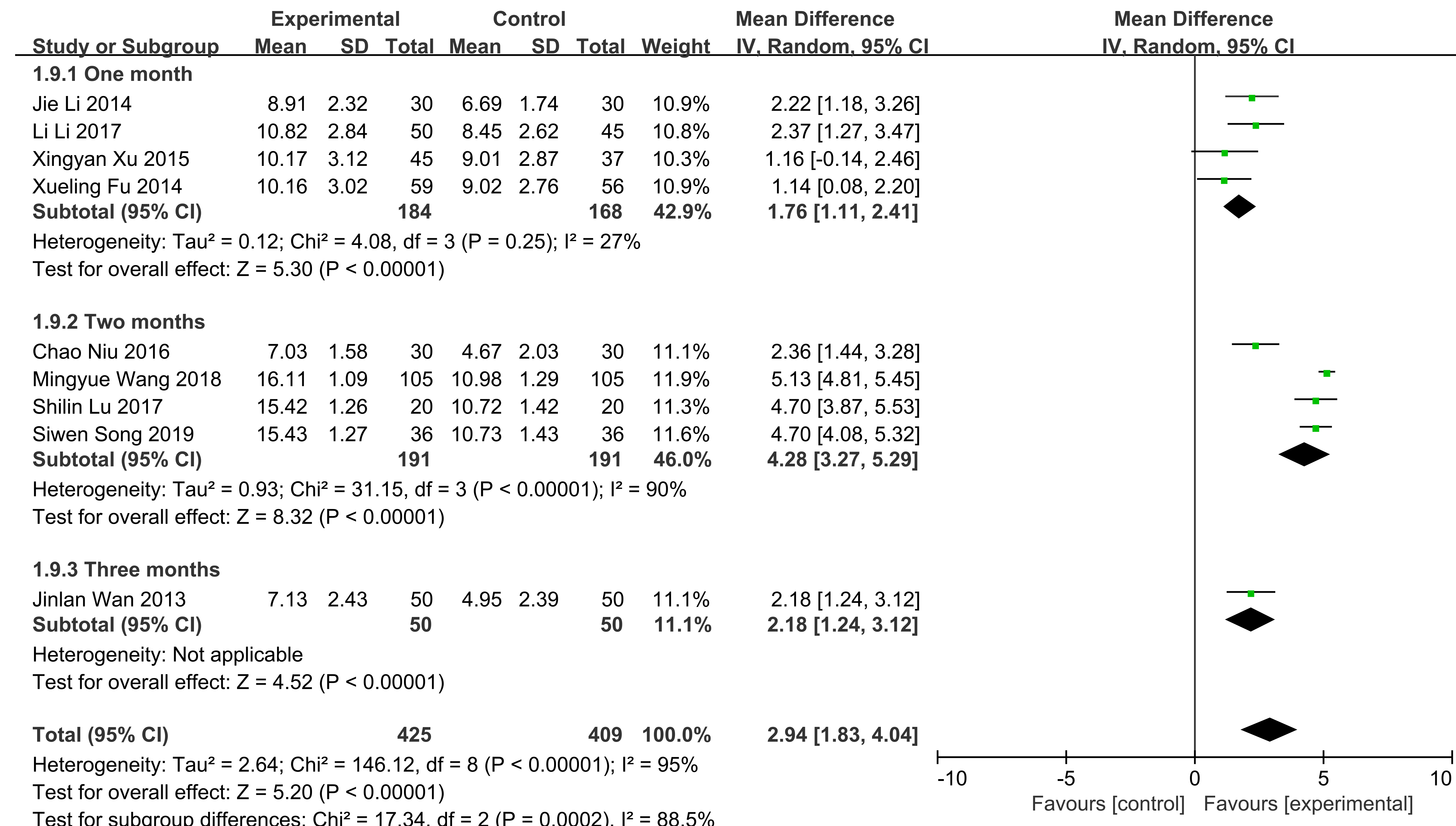

Supplement: Supplementary material 1 — The list of records excluded by reading the full text. [file DataSheet_1.zip › supplementary materials/supplementary material 11.tif]

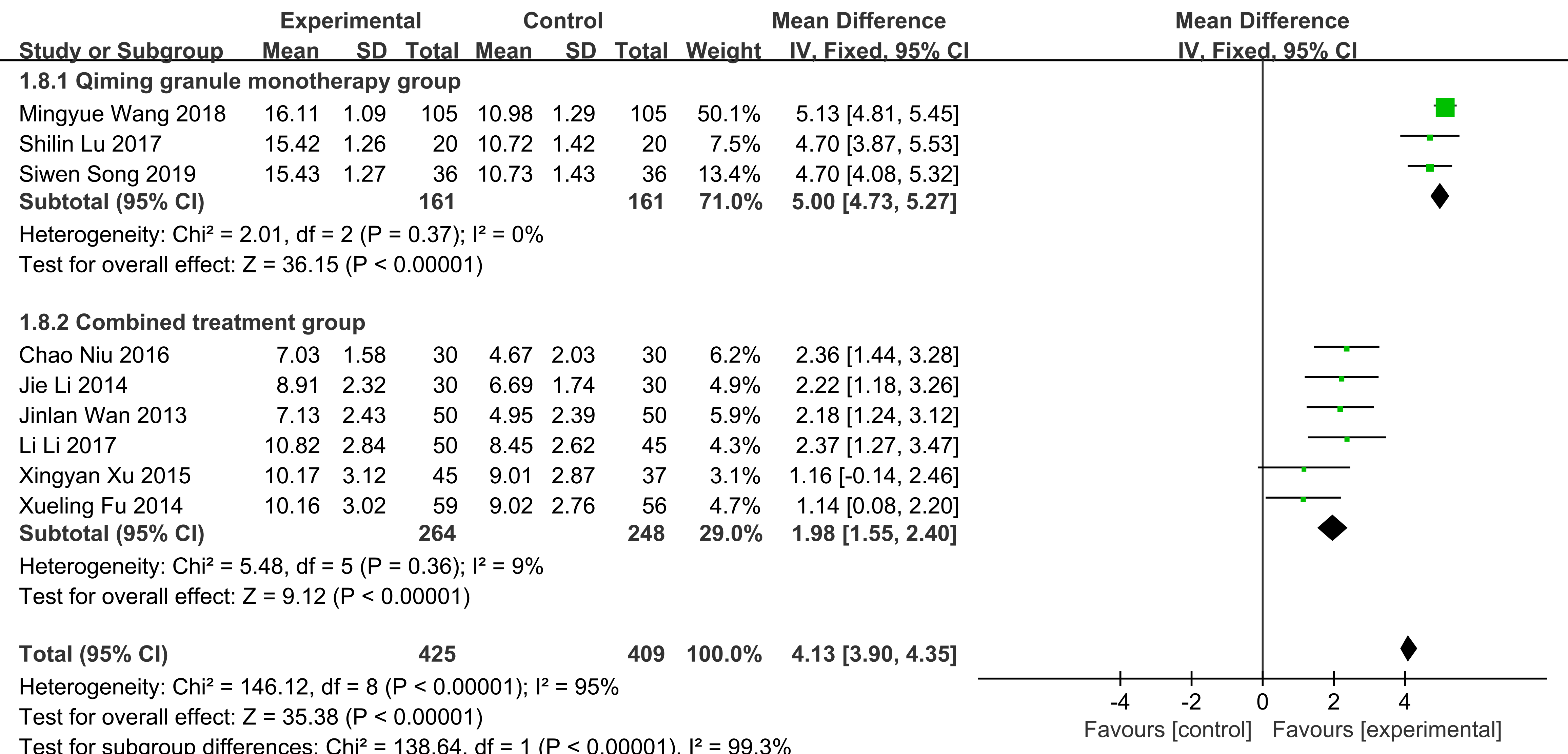

Supplement: Supplementary material 1 — The list of records excluded by reading the full text. [file DataSheet_1.zip › supplementary materials/supplementary material 12.tif]

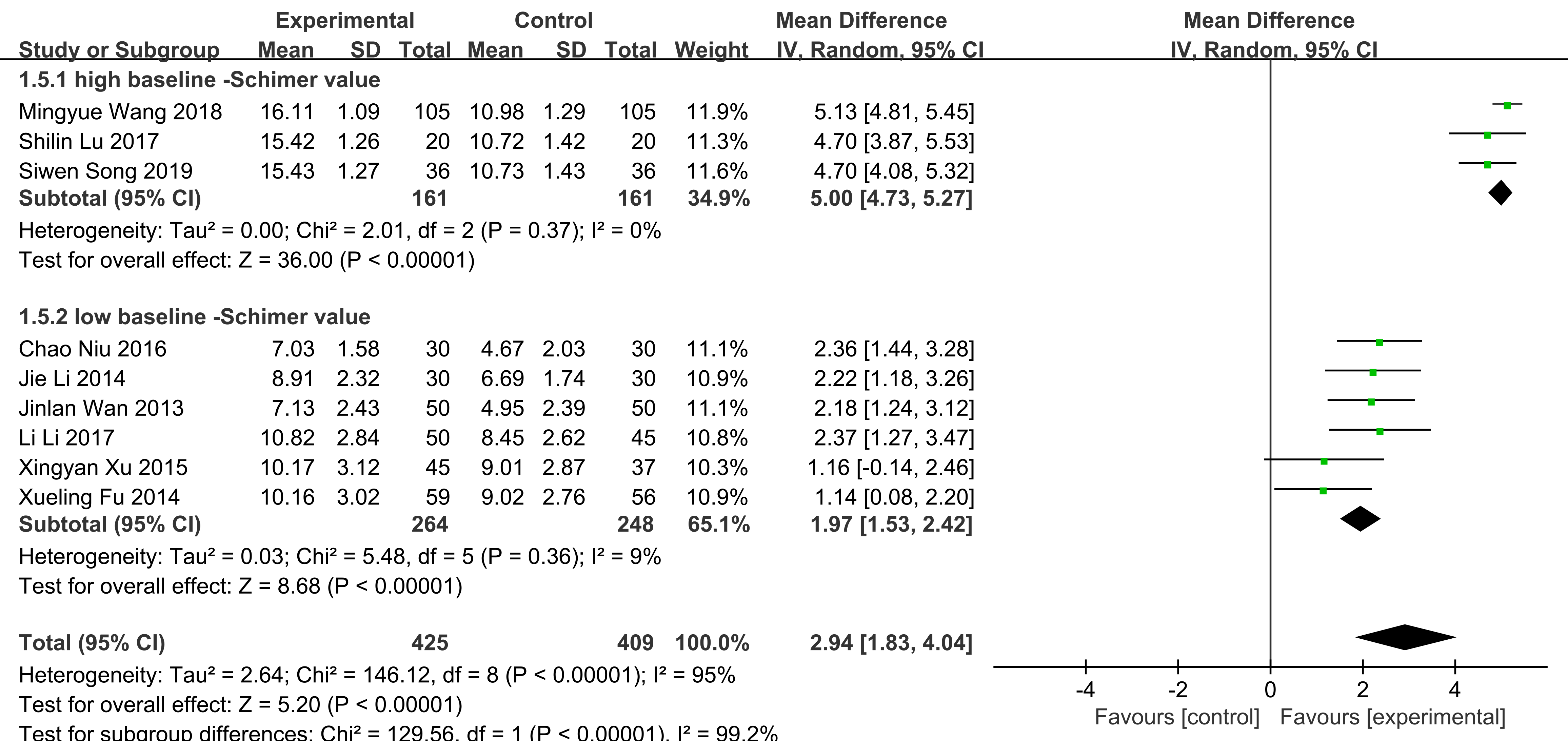

Supplement: Supplementary material 1 — The list of records excluded by reading the full text. [file DataSheet_1.zip › supplementary materials/supplementary material 13.tif]

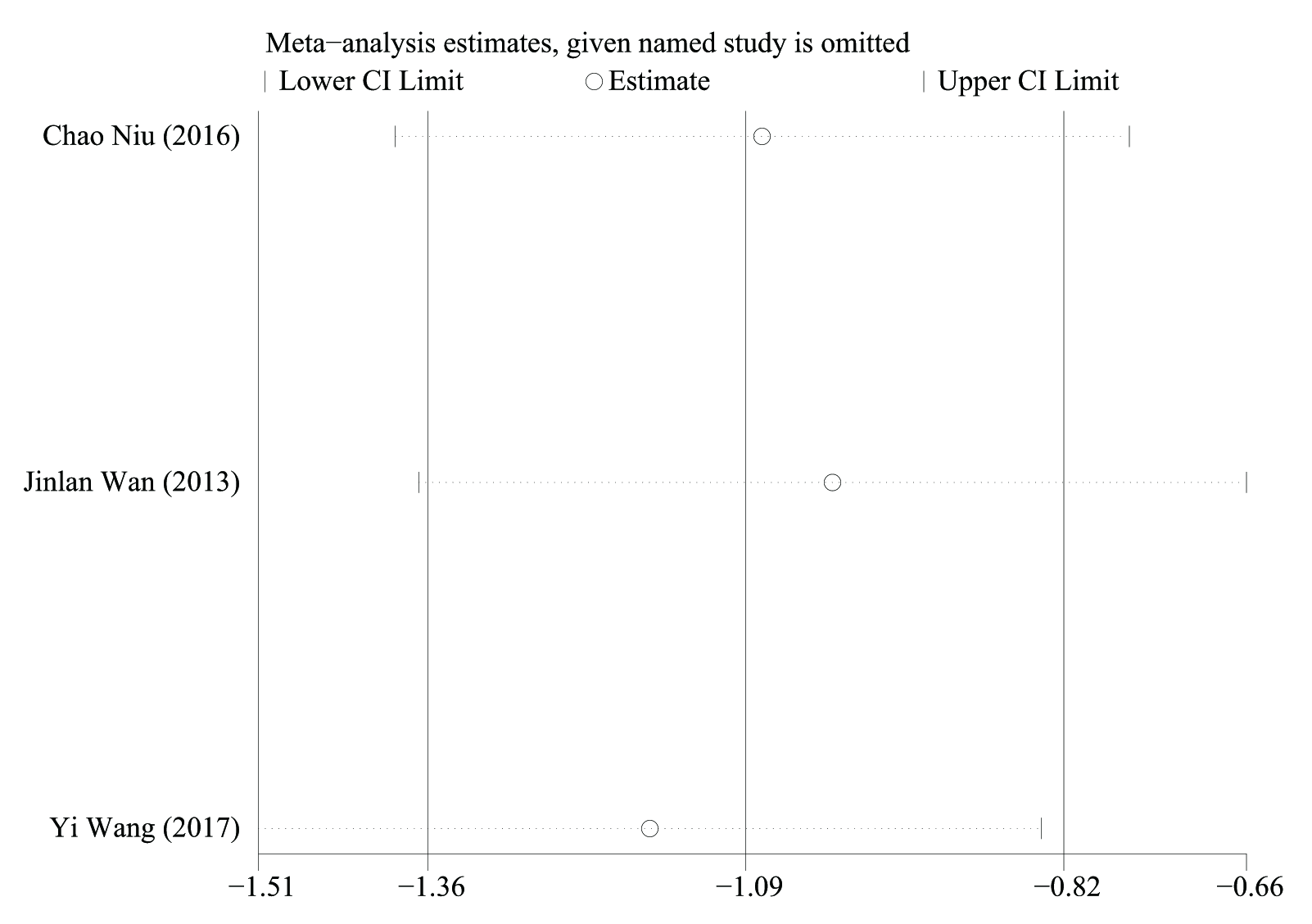

Supplement: Supplementary material 1 — The list of records excluded by reading the full text. [file DataSheet_1.zip › supplementary materials/Supplementary material 14.tif]

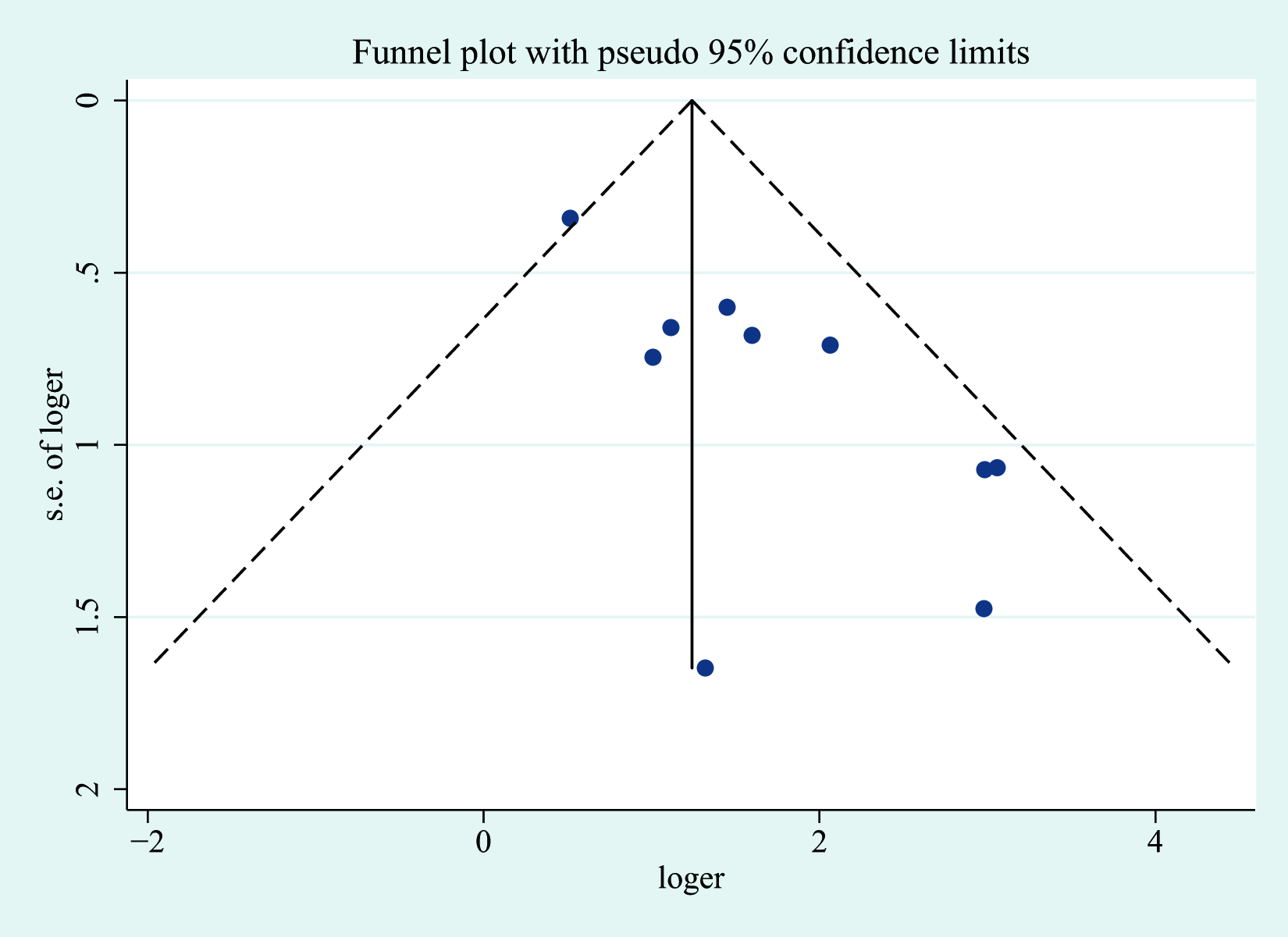

Supplement: Supplementary material 1 — The list of records excluded by reading the full text. [file DataSheet_1.zip › supplementary materials/Supplementary material 15.tif]

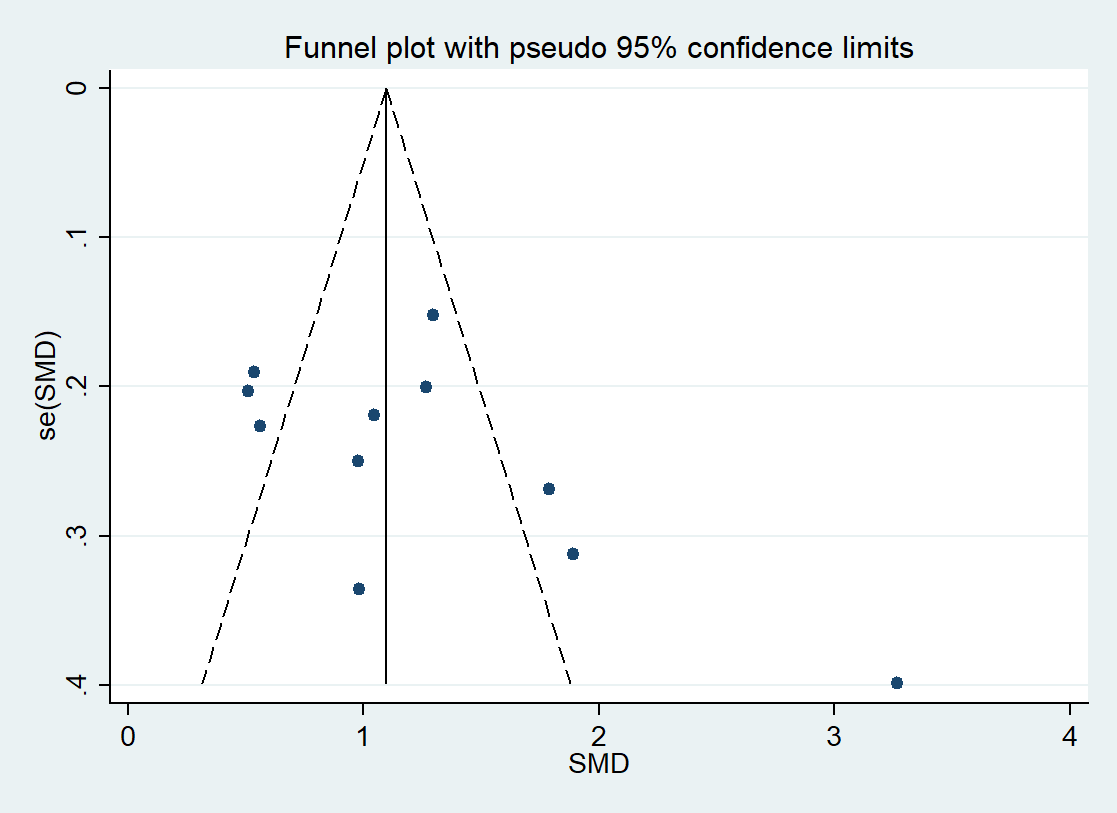

Supplement: Supplementary material 1 — The list of records excluded by reading the full text. [file DataSheet_1.zip › supplementary materials/Supplementary material 16.tif]

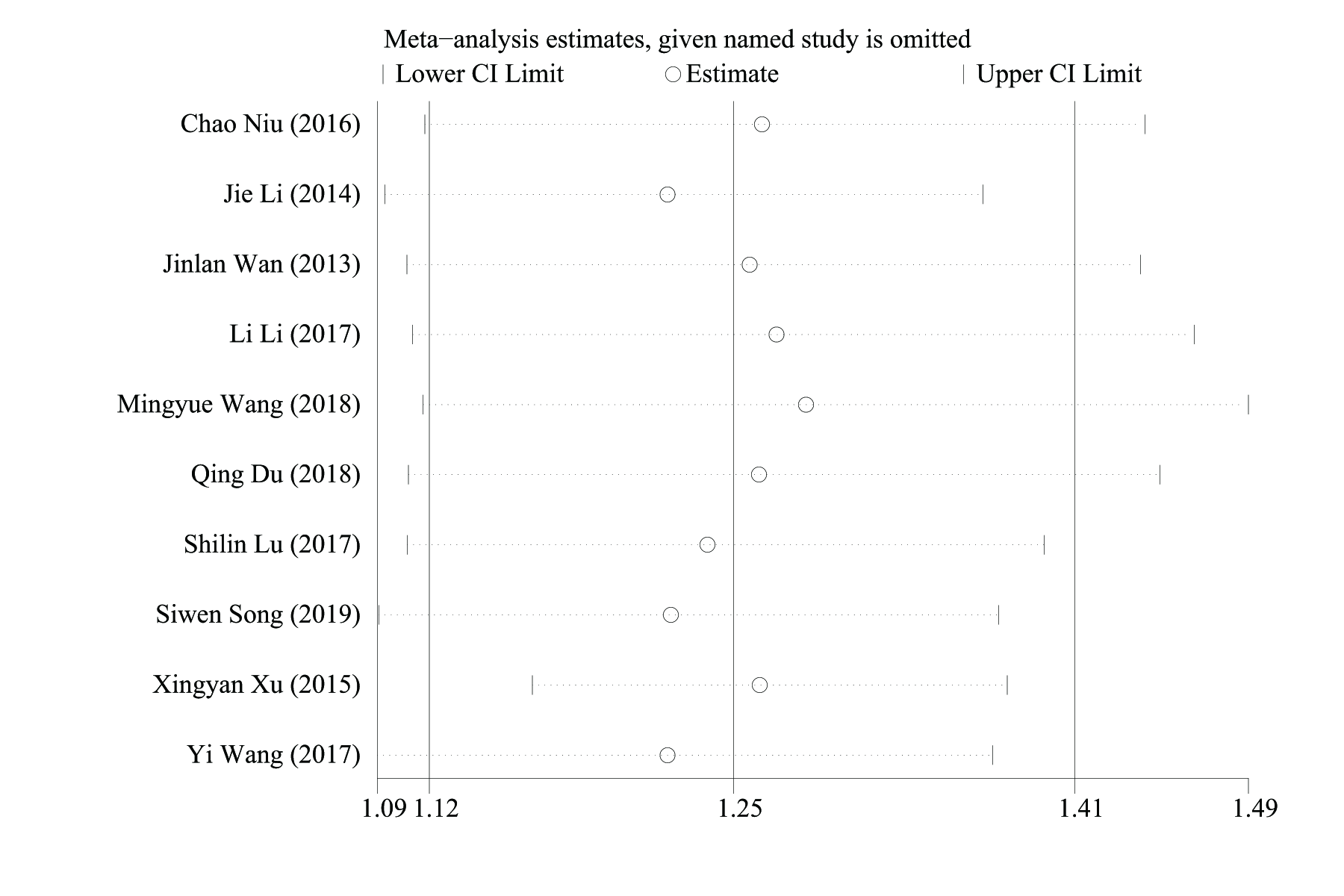

Supplement: Supplementary material 1 — The list of records excluded by reading the full text. [file DataSheet_1.zip › supplementary materials/Supplementary material 3.tif]

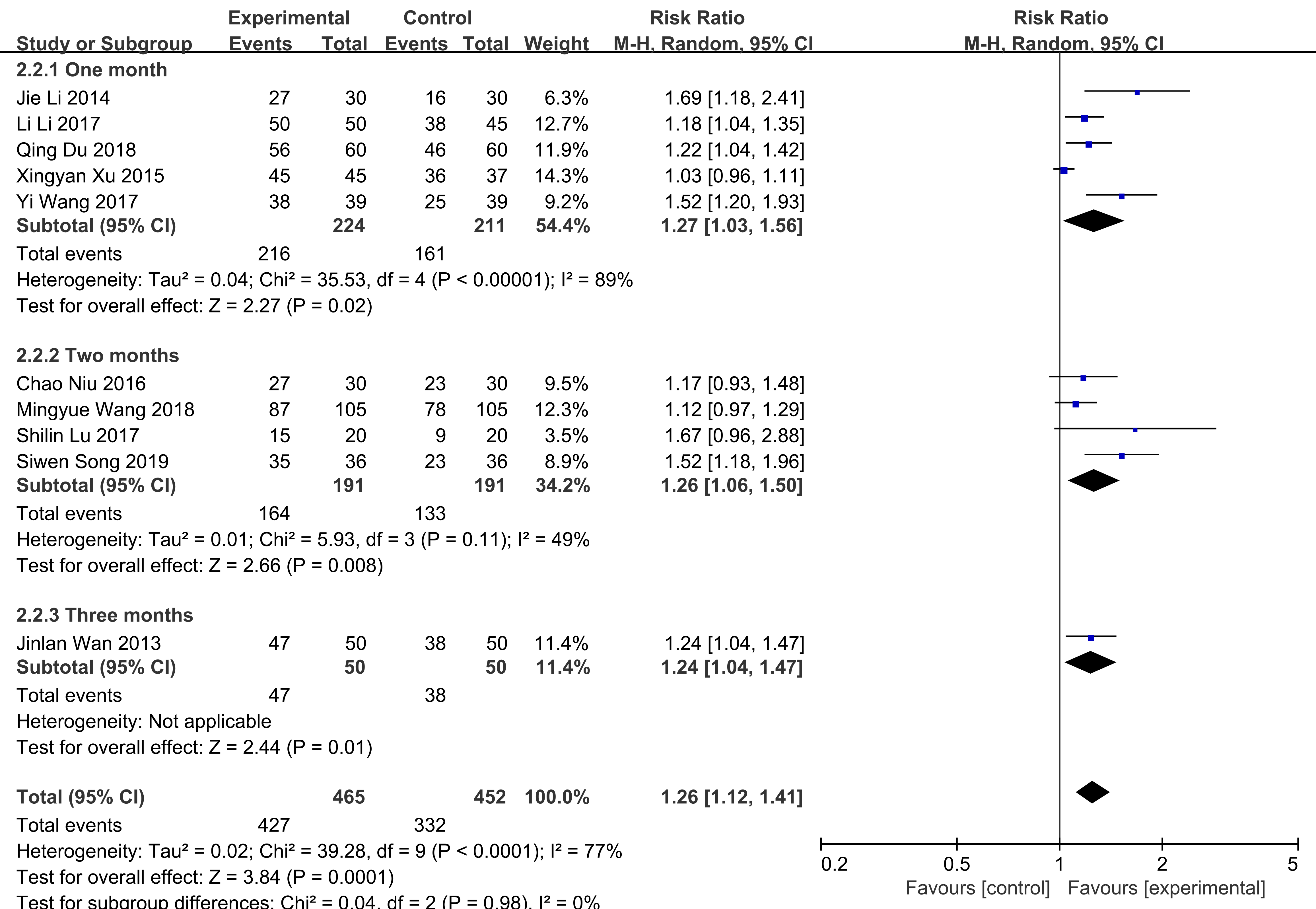

Supplement: Supplementary material 1 — The list of records excluded by reading the full text. [file DataSheet_1.zip › supplementary materials/Supplementary material 4.tif]

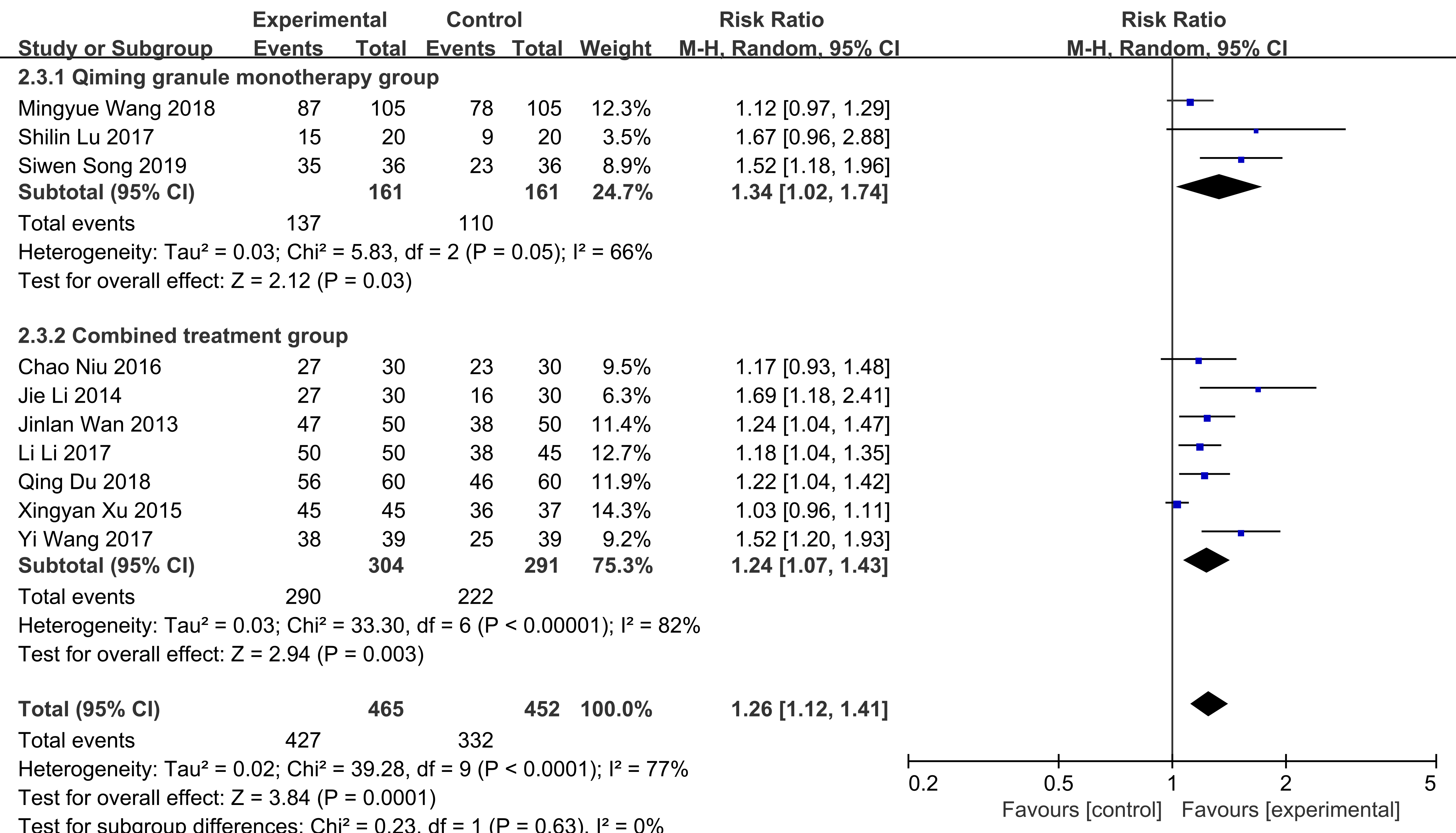

Supplement: Supplementary material 1 — The list of records excluded by reading the full text. [file DataSheet_1.zip › supplementary materials/Supplementary material 5.tif]

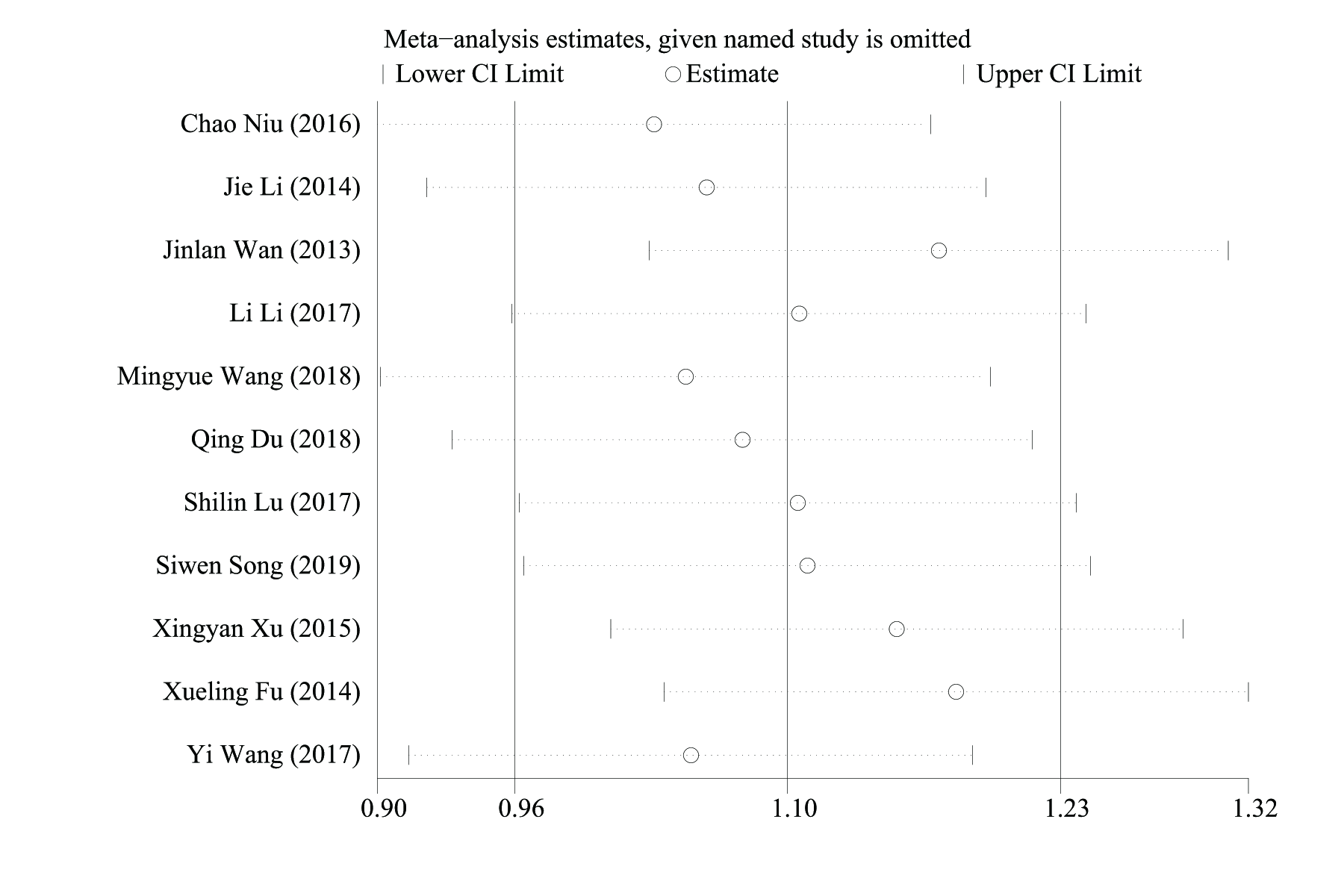

Supplement: Supplementary material 1 — The list of records excluded by reading the full text. [file DataSheet_1.zip › supplementary materials/Supplementary material 6.tif]

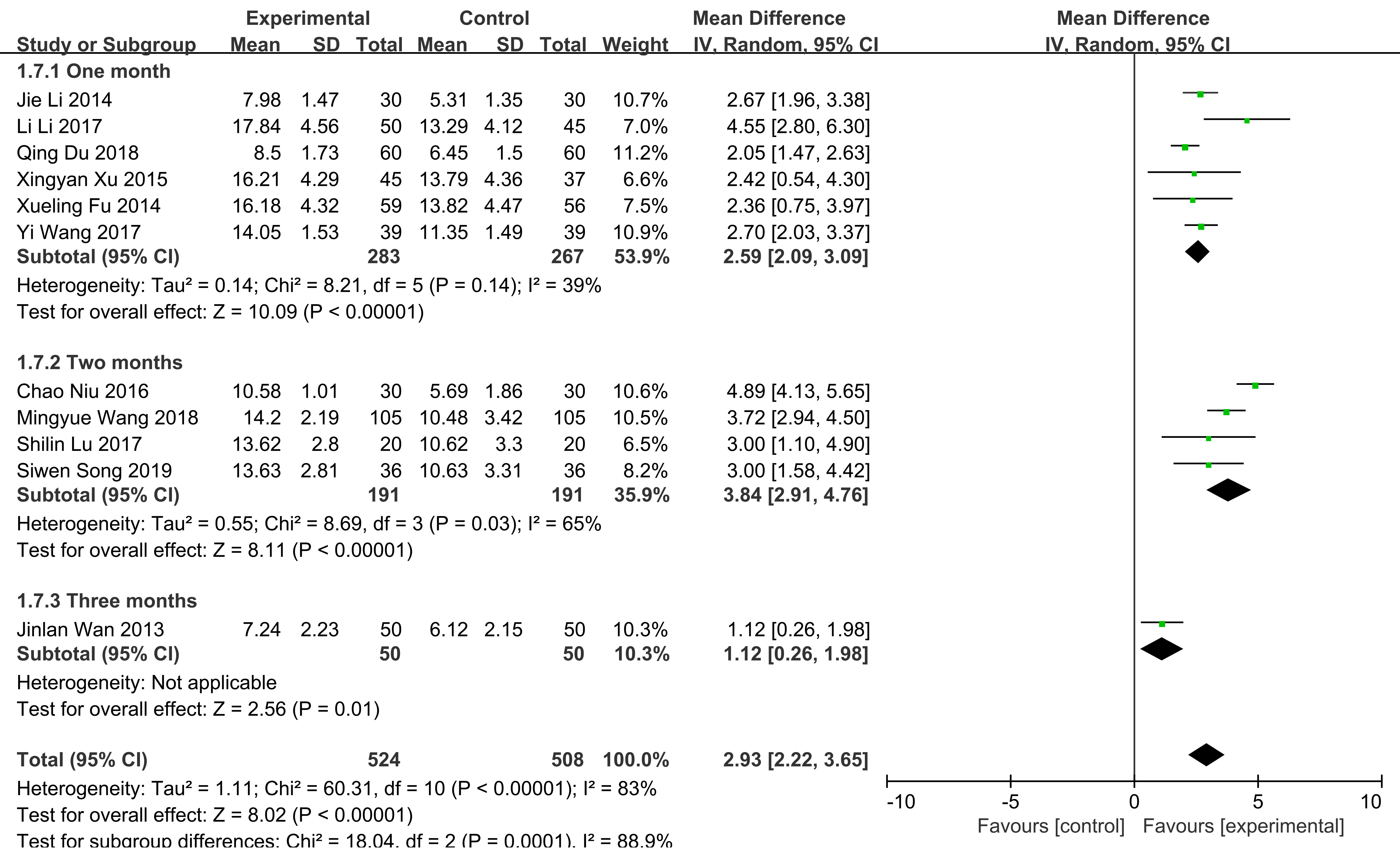

Supplement: Supplementary material 1 — The list of records excluded by reading the full text. [file DataSheet_1.zip › supplementary materials/supplementary material 7.tif]

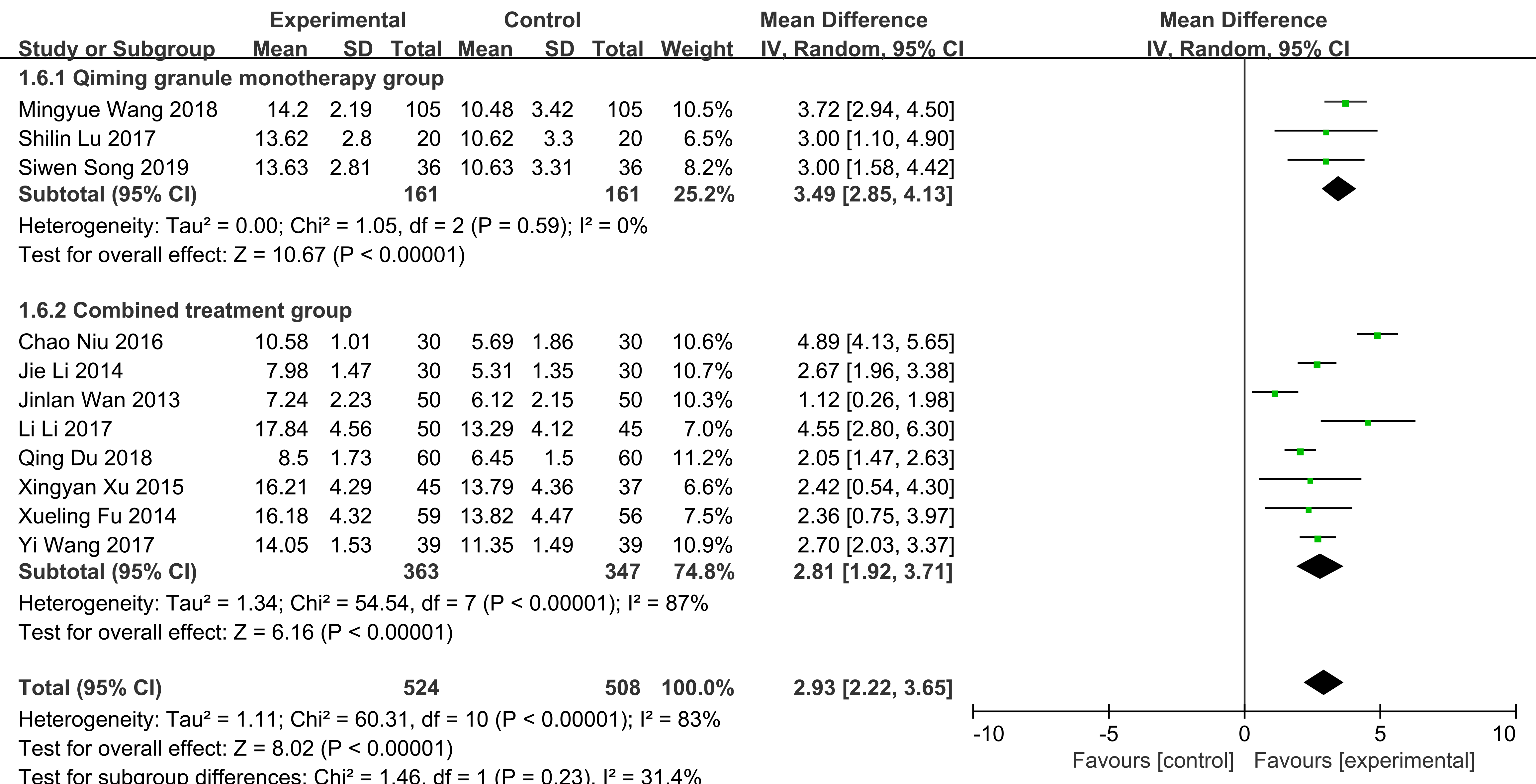

Supplement: Supplementary material 1 — The list of records excluded by reading the full text. [file DataSheet_1.zip › supplementary materials/supplementary material 8.tif]

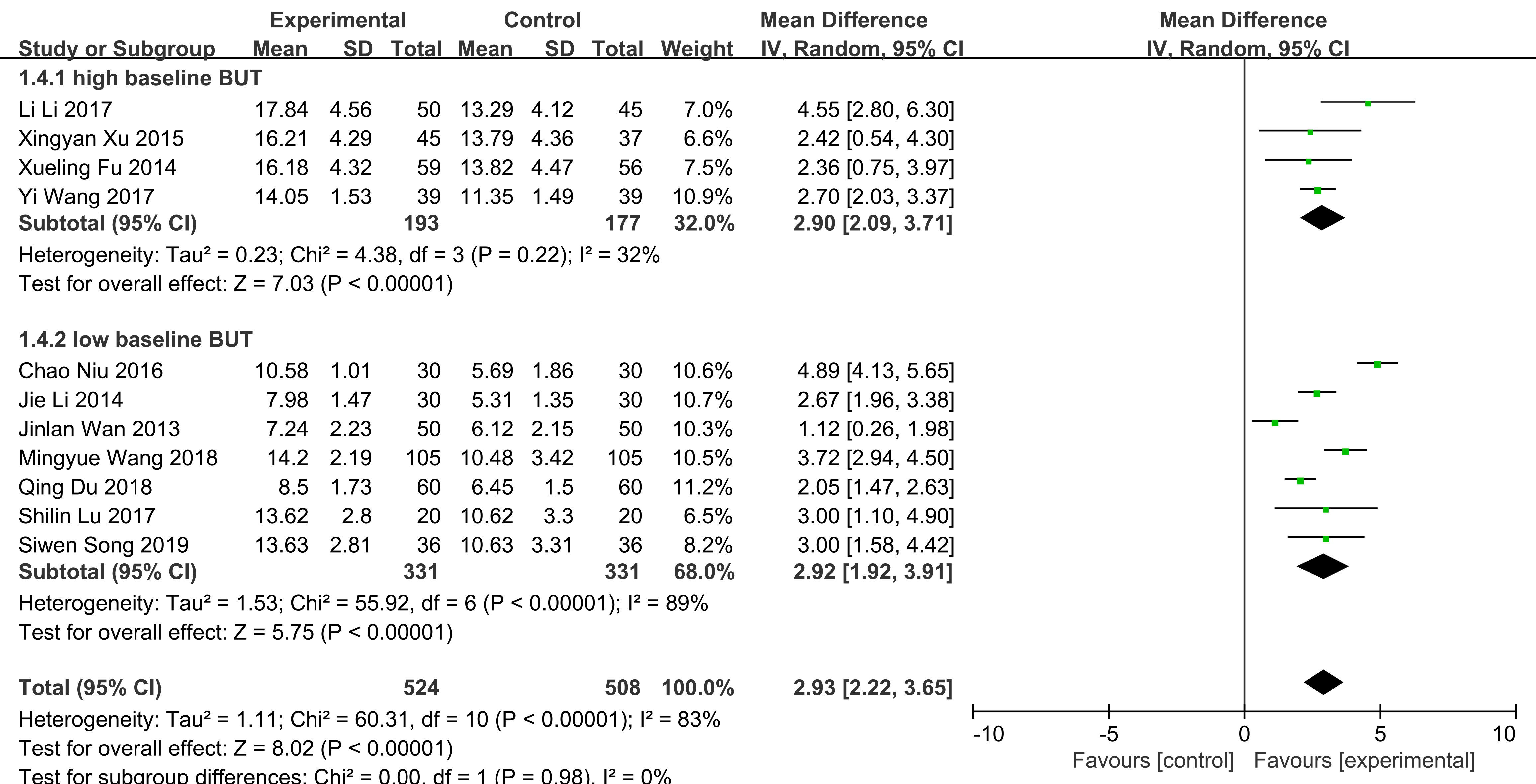

Supplement: Supplementary material 1 — The list of records excluded by reading the full text. [file DataSheet_1.zip › supplementary materials/supplementary material 9.tif]
